# A minor but deadly surgery of colonic polypectomy in an elderly and fragile patient: a case report and the review of literature

**DOI:** 10.1186/s12957-016-1010-6

**Published:** 2016-09-26

**Authors:** Xiaoming Yuan, Guangrong Zhou, Yan He, Aiwen Feng

**Affiliations:** 1 Department of Intestinal Surgery, Affiliated Huai’an First People’s Hospital, Nanjing Medical University, Huai’an, 223300 China; 2 Department of Oncological Radiotherapy, Affiliated Huai’an First People’s Hospital, Nanjing Medical University, Huai’an, 223300 China

**Keywords:** Colonic polyp, Hypoproteinemia, Anemia, COPD, Arrhythmias, Polypectomy, Intestinal leakage, Abdominal infection

## Abstract

**Background:**

Epithelial dysplasia and adenomatous polyps of colorectum are precancerous lesions. Surgical removal is still one of the important treatment approaches for colorectal polyps.

**Case presentation:**

A male patient over 78 years was admitted due to bloody stool and abdominal pain. Colonoscopic biopsy showed a high-grade epithelial dysplasia in an adenomatous polyp of sigmoid colon. Anemia, COPD, ischemic heart disease (IHD), arrhythmias, and hypoproteinemia were comorbidities. The preoperative preparation was carefully made consisting of oral nutritional supplements (ONS), blood transfusion, cardiorespiratory management, and hemostatic therapy. However, his illness did not improve but deteriorate mainly due to polyp rebleeding during preparative period. The open polypectomy was performed within 60 min under epidural anesthesia. Postoperative treatments included oxygen inhalation, bronchodilation, parenteral and enteral nutrition, human serum albumin, antibiotics, and blood transfusion. Unluckily, these did not significantly facilitate to surgical recovery on account of severe comorbidities and complications. The most serious complications were colonic leakage and secondary abdominal severe infection. The patient finally gave up treatment due to multiple organ dysfunction syndromes.

**Conclusions:**

The polypectomy for colonic polyp is a seemingly minor but potentially deadly surgery for patients with severe comorbidities, and prophylactic ostomy should be considered for the safety.

## Background

Adenomatous polyp and epithelial dysplasia are regarded as precancerous lesions of colorectal cancer [1]. Endoscopic intervention (e.g., EMR/ESD) and surgical removal (e.g., polypectomy/colectomy) are important management approaches [1–3]. In general, surgical removal should be considered where the malignancy is suspected or concerns about the likelihood of incomplete endoscopic resection or endoscopic contraindications [2, 4]. The limited surgical resection is fit for patients with comorbidities [2], but prophylactic ostomy is not explicitly elaborated in many literatures [1–5]. In this case, we reported a case of deadly colonic leakage after laparotomic polypectomy for an adenomatous polyp of colon.

## Case presentation

A male patient over 78 years was admitted on Sep. 9, 2015, due to bloody stool and abdominal pain. Past history included smoking, bronchitis, COPD, but no cirrhosis and nephrotic syndrome. Physical exam showed normal vital signs, lung hyperresonance, and scattered crackles. The abdomen was flat without GI pattern. Tenderness and rebound tenderness were negative. Right indirect hernia existed where gut were palpable. Mass, anal fissure, and hemorrhoid were not found in the rectum and anus. Lung function showed obstructive ventilation dysfunction (Table 1). ECG and Doppler echocardiography showed arrhythmias, ischemic heart disease (IHD), pulmonary hypertension, and reduced left ventricular compliance (Table 1). The heart function classification was II. Colonoscopy revealed a pedunculated and easy bleeding polyp of sigmoid colon, but did not find ischemic bowel disease, IBD, and diverticula. A biopsy for polyp samples showed high-grade epithelial dysplasia and adenomatous polyp (Table 1). CT scan showed emphysema, right hernia where gut could be watched, prostatic hyperplasia, and hepatorenal cysts, but no visible masses in GI tract (Table 1). Laboratory findings showed anemia, positive FOBT, and hypoproteinemia. The coagulation, hepatorenal function, and arterial blood gas were almost normal (Table 2). APACHE II score was 10 points. ASA classification grade was III. Goldman Cardiac Risk Index score and European Nutritional Risk Screening (NRS 2002) score were 15 and 3 points, respectively.

**Table 1:** The examination findings before and after colonic polypectomy

Item	Examination findings
ECG	Sinus tachycardia, frequent premature atrial complexes, occasional ventricular premature beat, ischemic ST-T change
CUS	Moderate pulmonary arterial hypertension, mild aortic valvular regurgitation, reduced left ventricular compliance
CT	Bilateral emphysema, right indirect inguinal hernia, prostatic hyperplasia, hepatorenal cysts, normal esophagus/gastrointestine/colon, but suspicious thickness of the upper rectum
PF	Severe mixed ventilation dysfunction, MVV 20 L/min (24 % of predicated value), FEV1 0.72 (34 % of predicated value)
CS	A pedunculated neoplasm (3.0 × 2.0 × 1.5 cm in size, villous and friable and easy bleeding) of sigmoid colon
POB	Colonic villioustublar adenoma in accompany with epithelial high-grade dysplasia
POP	Colonic villioustublar adenoma in accompany with epithelial high-grade dysplasia and focal canceration

*ECG* electrocardiography, *CUS* cardiac ultrasound, *CT* computed tomography, *PF* pulmonary function, *MVV* maximum ventilator volume, *FEV1* forced expiratory volume in first second, *CS* colonoscopy, *POB* preoperative biopsy, *POP* postoperative pathology

**Table 2:** Laboratory examinations before and after colonic polypectomy

Time	Blood routine													Coagulation function					
	WBC (10^9^/L)	RBC (10^12^/L)	Hb (g/L)	PLT (10^9^/L)		Hct (%)		TL (10^9^/L)		NR (%)		Lym (%)		PT (s)	APTT (s)	TT (s)	INR		d-dimer (μg/mL)
09/09	4.82	2.66	80	123		26.5		0.88		71.3		18.3		11.9	31.3	17.2	0.89		–
18/09	5.17	2.88	86	137		28.5		1.08		66.8		20.9		–	–	–	–		–
24/09	7.70	2.95	94	132		28.8		0.81		85.3		10.5		–	–	–	–		–
27/09	4.41	2.60	78	56		25.5		0.44		83.2		10.1		–	–	–	–		2.22
30/09	7.07	2.78	84	75		26.6		0.70		81.4		9.9		–	–	–	–		–
03/10	6.57	3.11	94	75		29.5		0.46		89.4		7.0		–	–	–	–		1.07
07/10	3.16	2.83	85	27		27.0		0.45		78.1		14.1		–	–	–	–		–
Time	Blood biochemistry													Arterial blood gas					
	ALB (g/L)	PALB (mg/L)	ALT (U/L)	AST (U/L)	Urea (mM)		Cr (μM)		K^+^ (mM)		Na^+^ (mM)		Cl^−^ (mM)	pH	PaCO_2_ (mmHg)	PaO_2_ (mmHg)	SaO_2_ (%)	LA (mM)	
09/09	31.6	134.0	6.0	11.0	6.99		58.0		4.84		142.4		102.8	–	–	–	–	–	
18/09	28.3	146.0	10.0	18.0	–		–		4.65		144.5		106.0	7.440	47.9	88.2	97.0	0.7	
24/09	24.6	74.0	10.0	16.0	5.74		71.0		4.15		144.4		108.0	–	–	–	–	–	
27/09	25.5	44.0	11.0	39.0	–		–		4.42		144.9		107.7	–	–	–	–	–	
30/09	25.6	44.0	8.0	34.0	13.73		64.0		3.13		143.2		99.3	–	–	–	–	–	
03/10	24.5	19.0	7.0	20.0	23.51		147.0		4.09		139.7		97.0	7.435	36.8	118.0	98.8	1.8	
07/10	25.5	15.0	11.0	20.0	29.43		74.0		3.70		148.3		108.4	7.24	55.4	83.2	92.7	2.4	

*WBC* white blood cell, *RBC* red blood cell, *Hb* hemoglobin, *PLT* platelet, *Hct* hematocrit, *TL* total lymphocyte, *NR* neutrophil ratio, *Lym* lymphocyte, *PT* prothrombin time, *APTT* activated partial thromboplastin time, *TT* thrombin time, *INR* international normalized ratio, *Fn* fibrinogen, *ALB* albumin, *PALB* pre-albumin, *ALT* alanine aminotransferase, *AST* aspartate aminotransferase, *Cr* creatinine, *LA* lactic acid

A 2-week preoperative preparation was made. Bronchitis and COPD were managed by stopping smoking, low-flow oxygen therapy, blowing balloon exercise, and administration of aminophylline and levofloxacin. Anemia was treated by blood transfusion of 4 U PRBCs, bleeding by vitamin K, and arrhythmias by β-receptor blocker. Malnutrition was treated by amino acid, dextrose, fatty emulsion, vitamins, and trace elements, as well as oral bifidobacteria and peptison based on dietary supplement. After treatment, lung scattered rales completely resolved. Nevertheless, anemia and hypoproteinemia did not significantly improve but deteriorate due to rebleeding from colonic polyp. Thus, laparotomic polypectomy was performed under epidural anesthesia on Sep. 23, 2015. An incision of the colonic wall was longitudinal and was sutured transversely. The operative time was 60 min and blood loss was little. Early postoperative management included respiratory management, restrictive fluid administration, combined antibiotics, octreotide, and parenteral nutrition (25–30 kcal/kg/day). Human serum albumin was given at a dose of 10 g daily. During the first 3 days, the patient was uneventful and oral fluid diet containing ONS was provided after anal aerofluxus. At the fourth day, he suffered from acute heart dysfunction (AHD) diagnosed by manifestations of fatigue, dyspnea, oliguria, and pulmonary rales. ECG monitor showed normal BP, decreased SaO_2_%, and increased heart rate. The CVP value was >15cmH_2_O. Re-assessment of cardiac function classification was IV. The clinical features of AHD rapidly resolved after injection of cardiotonic and diuretic. Laboratory test showed normal electrolytes and decreased Hb level (78 g/L) (Table 2), so 1 U PRBCs were transfused. At the 7th day laboratory findings revealed abnormal renal function (Table 2). At the 10th day, he complained of abdominal distension and dramatic increase in volume of right hernia. B ultrasound revealed pleural effusion and massive ascites. Laboratory findings revealed worse renal function (Table 2). At the 11th day, he was re-struck by AHD, which was also rapidly corrected by cedilanid and furosemide. Massive pale yellow ascites outflowed from abdominal incision and abdominal drainage tube. At the 12th day, he had colonic leakage as judged by intestinal content outflowing from abdominal incision and drainage tube, which had drained out little pale bloody fluid and pale yellow ascites during past 11 days. At the 14th day, he gave up treatment due to severe infection and MODS (Table 2).

## Discussion

Epithelial dysplasia and tumorous polyps are regarded as precursor lesions of large bowel carcinoma. The risk of adenomatous polyp is significantly increased in COPD patients [6]. In clinic, most polyps can be treated by EMR and ESD, and only a few cases need surgery [7]. In this case, the patient was not a good candidate for endoscopic management according to ESGE guideline [8]. A preparation was made according to preoperative evaluation including smoking history, cardiopulmonary function, nutritional status, and ASA classification. Unluckily, this preparation was depressing because anemia and malnutrition (hypoproteinemia and reduced peripheral blood lymphocyte) did not improve but deteriorate (Table 2), which was associated with uncontrollable rebleeding from polyp. Thus, this polypectomy to some extent was done under a potential risk circumstance.

In order to minimize trauma, we selected small median incision and epidural anesthesia [9–11]. This polypectomy was performed in a short time. Postoperative measures were taken such as provision of nutritional support, human serum albumin, antibiotics, and cardiopulmonary management. Unluckily, severe comorbidities and complications including acute heart dysfunction, anemia, renal dysfunction, and malnutrition disturbed sickness recovery. The most serious complications were colonic leakage and severe abdominal infection, which generally carry a high mortality [12].

COPD is an irreversible and chronic inflammatory pulmonary disorder [13], and 25–40 % patients with COPD are reported to be undernourished [14–16], as is referred to lung cachexia [13]. COPD and malnutrition are confirmed to be predictive markers for poor prognosis and high mortality [13, 17, 18]. The most important mechanism for COPD-induced malnutrition is the mismatching between protein biosynthesis and breakdown [14–16]. The adverse factors for malnutrition include disordered hormones [19], inflammatory cytokines (e.g., IL-6, TNFα [20], CRP [21]), cigarette smoking [22], poor physical activity [19], and hypoxemia [14, 19]. Further, serum total protein ≤55 g/L and albumin ≤35 g/L are predictors for gut leakage [23, 24]. In this case, severe COPD and malnutrition hypoproteinemia (Table 2) played an important role in leakage generation.

Age and gender are also independent risk factors for gut leakage. It is reported that the morbidity of gut leakage in patients ≥65 years is 1.31-fold compared to those <65 years [12]. Studies have further showed that elderly patients ≥75 years [25] usually have reduced whole function reserve, more internal chronic diseases, worse operative tolerance, slower postoperative recovery, and higher complication and mortality rate [26, 27]. A prospective study by Kotoč et al. shows that the morbidity of anastomotic leakage in patients with sphincter-saving rectal resections is 10.9 %, and these patients all are males [18]. Another study report by Nasirkhan shows the morbidity of gut leakage in male patients (13.4 %) was significantly higher than that in female patients (5.2 %) [28]. In this case, the elderly age and male gender were harmful factors for leakage formation.

Anemia is a common comorbidity in patients with gastrointestinal disease, especially malignancy. Some studies suggested anemia is a negative factor for postoperative recovery. Hemoglobin ≤94 g/L is an independent adverse prognostic factor for leakage [23]. Thus, correction of anemia constitutes an important component of perioperative managements. However, blood transfusion in turn may decrease body’s immunocompetence [12]. It has been documented that blood transfusion of >2 U PRBCs may contribute to intestinal leakage generation [22, 24]. Though these studies are all retrospective, further prospective studies we think are still needed. In this case, anemia and blood transfusion of 5 U PRBCs were likely involved in facilitating to gut leakage.

Cigarette smoking has a detrimental action on healing of surgical wounds [29]. Tobacco consists of at least three toxic substances: nicotine, tar, and carbon monoxide. Studies have demonstrated that these toxins can contract blood vessels and reduce blood supply, accompanying with decrease in contractility of vessels, blood flow rate, and efficiency of oxygen transportation [30]. In addition, smoking may give rise to a significant decline in body’s resistance to infection [30]. Recently, a large prospectively collected clinical database has indicated that smoking is associated with an increased risk of leakage [31]. In this case, long-term cigarette smoking inducing vessel contraction and hypoxia might play a role in disunion of colon-wall incision.

Cardiac dysfunction is closely associated with COPD, IHD, and volume overload. COPD and IHD are mutually influenced. Patients with COPD and IHD show worst outcomes compared to those with only COPD or IHD [32], as shown by an increase in risk of heart failure, acute exacerbation of COPD, and mortality [32, 33]. The mechanism of volume overload in COPD patients was the activation of sodium-retaining mechanisms [34]. COPD patients with hypoalbuminemia are also prone to develop acute heart dysfunction [35]. In addition, premature ventricular complexes have an adverse influence on pump function [36]. In this case, these factors such as COPD, IHD, hypoproteinemia, and arrhythmia to some extent participated in inducing acute heart dysfunction.

Epidural anesthesia is generally regarded as to be safe in many surgical operations [9–11]. Epidural anesthesia is observed to increase motor activity in the small bowel as well as left-sided colon and rectum. Tonic and segmental contractions are recorded from the sigmoid colon to rectum [37]. The increase of motor activity is often deemed as a marker of gut function recovery. However, increased motor activity may expose a constructed anastomosis to undue strain in the postoperative period [37], which is a detrimental factor to the healing of anastomosis [24]. In addition, it has been recently reported that an ASA score of ≥3 is related to an increased risk of gut leakage [31]. In this case, the patient with a ASA score of 3 had a fast recovery of gut peristaltic function after epidural anesthesia, but this increased motor activity was also an adverse factor for incision healing of the sigmoid colon.

Prophylactic ostomy is used in certain operations for colorectal cancer [38, 39] and non-neoplasm diseases such as familial adenomatous polyposis and ulcer colitis [40, 41]. Prophylactic ostomy can effectively prevent feces from inflowing into abdominal. However, prophylactic ostomy is not a routine surgical procedure for treating a single colorectal polyp. Thus, in this case, the patient was only done polypectomy without prophylactic colostomy. Unluckily, his multiple comorbidities and postoperative complications severely interfered with surgical incision healing, resulting in deadly colonic leakage and secondary diffuse abdominal infection.

## Conclusions

Laparotomic polypectomy is a minor but potentially deadly operation for the very old and fragile patients with severe comorbidities, and prophylactic ostomy should be considered for the safety.

## Abbreviations

AHD: Acute heart dysfunction
COPD: Chronic obstructive pulmonary disease
IBD: Inflammatory bowel disease
MODS: Multiple organ dysfunction syndrome
ONS: Oral nutritional supplements
PRBCs: Packed red blood cells
